# Isolation and characterization of antagonistic *Paenibacillus polymyxa* HX-140 and its biocontrol potential against Fusarium wilt of cucumber seedlings

**DOI:** 10.1186/s12866-021-02131-3

**Published:** 2021-03-06

**Authors:** Yang Zhai, Jiu-xiang Zhu, Tai-meng Tan, Jian-ping Xu, Ai-rong Shen, Xie-bin Yang, Ji-lie Li, Liang-bin Zeng, Lin Wei

**Affiliations:** 1 Institute of Bast Fiber Crops, Chinese Academy of Agricultural Sciences, No.348 Xianjiahu West Road, Changsha, 410205 Hunan China; 2 Hunan Provincial Key Laboratory of Forestry Biotechnology, Central South University of Forestry and Technology, Changsha, 410004 China; 3 Hunan Academy of Forestry, Changsha, 410004 China; 4 Institute of Plant Protection, Hunan Academy of Agricultural Sciences, No.726 Yuanda 2nd Road, Changsha, 410125 Hunan China

**Keywords:** Fusarium wilt, Cucumber, *Paenibacillus polymyxa*, Antagonistic bacterium, Biocontrol mechanism

## Abstract

**Objective:**

The aim of this study is to evaluate the efficacy of the strain *Paenibacillus polymyxa* HX-140, isolated from the rhizosphere soil of rape, to control Fusarium wilt of cucumber seedlings caused by *Fusarium oxysporum* f. sp. *cucumerinum*.

**Results:**

Strain HX-140 was able to produce protease, cellulase, β-1,3-glucanase and antifungal volatile organic compounds. An in vitro dual culture test showed that strain HX-140 exhibited broad spectrum antifungal activity against soil-borne plant pathogenic fungi. Strain HX-140 also reduced the infection of Fusarium wilt of cucumber seedlings by 55.6% in a greenhouse pot experiment. A field plot experiment confirmed the biocontrol effects and further revealed that antifungal activity was positively correlated with inoculum size by the root-irrigation method. Here, inoculums at 10^6^ 10^7^ and 10^8^ cfu/mL of HX-140 bacterial suspension reduced the incidence of Fusarium wilt of cucumber seedling by 19.5, 41.1, and 50.9%, respectively.

**Conclusions:**

Taken together, our results suggest that *P. polymyxa* HX-140 has significant potential in the control of Fusarium wilt and possibly other fungal diseases of cucumber.

**Supplementary Information:**

The online version contains supplementary material available at 10.1186/s12866-021-02131-3.

## Background

Cucumber is an economically important and popular vegetable crop. Vascular wilt of cucumber caused by *Fusarium oxysporum* f. sp. *cucumerinum* J. H. Owen (FOC) is one of the most destructive soil-borne diseases that causes large economic losses on a worldwide scale [33]. Pathogens of the *Fusarium* genus have an extremely broad host range including cotton, tomato, banana, strawberry, potato, capsicum, beans, peas, chickpea, and melons, and are among the most harmful soil-borne pathogens in crop production systems. They usually infect plants through the roots, causing damping-off, root rot, and vascular wilt [9, 44].

Chemical control using fungicides has been the most common control strategy for managing diseases caused by *Fusarium* spp. However, their extensive usage has not only caused negative environmental and toxicological impact but has also jeopardized human and animal health [44]. Furthermore, the application of chemical fungicides is known to select for resistant strains which limits their long-term effectiveness [19, 28]. Therefore, efficient and safe methods to prevent and control diseases caused by Fusarium wilt are urgently needed.

Biological control is an ecologically friendly, sustainable, and safe method for the control of plant infections. Biocontrol is a reliable alternative to the use of chemical fungicides [15]. Specifically, biocontrol of Fusarium wilts via bacterial antagonists has been extensively studied. For example, *Bacillus subtilis* SQR9 was found to control Fusarium wilt of cucumber by colonizing plant roots and producing lipopeptides (fengycin and bacillomycin) that were effective against *F. oxysporum* [8]. *B. velezensis* RC 218 reduced disease severity of Fusarium head blight (FHB) caused by *Fusarium graminearum* and reduced the accumulation of the associated mycotoxins [31]. “Amfissis” trees treated with *Paenibacillus Alvei* K165 exhibited significantly lower disease severity and lower relative AUDPC (Area Under the Disease Progress Curve) values than those of un-treated, diseased plants [27]. *Streptomyces griseorubens* E44G was found to possess chitinolytic activity and was capable of decreasing the severity of Fusarium wilt of tomato and increasing the growth and yield of tomato [35]. Lastly, isolates of the fungus *Trichoderma asperellum* that exhibited high levels of chitinase and β-1,3-glucanase activities strongly inhibited the mycelia growth of *F. oxysporum* f. sp. *Lycopersici* [14].

In the context of the current study, the bacterium *Paenibacillus polymyxa* has shown strong inhibitory effects against pathogenic nematodes, oomycetes, and fungi, including *F. oxysporum*. This biocontrol agent was reported to produce antifungal peptides, volatiles, proteins, and other antimicrobial substances that inhibit fungal pathogen mycelial growth. Several studies have revealed multiple mechanisms of anti-fungal activities, including competition for nutrients, the production of antifungal compounds such as antibiotics, extracellular enzymes, and organic volatiles [16] and the induction of plant resistance.

In this study, an antagonistic bacterium (*P. polymyxa* isolate HX-140) with antagonistic effects on FOC and other phytopathogenic fungi was identified, and its potential mechanism and control effect of Fusarium wilt disease were also explored.

## Results

### Screening and characterization of HX-140 strain

**Fig. 1:**
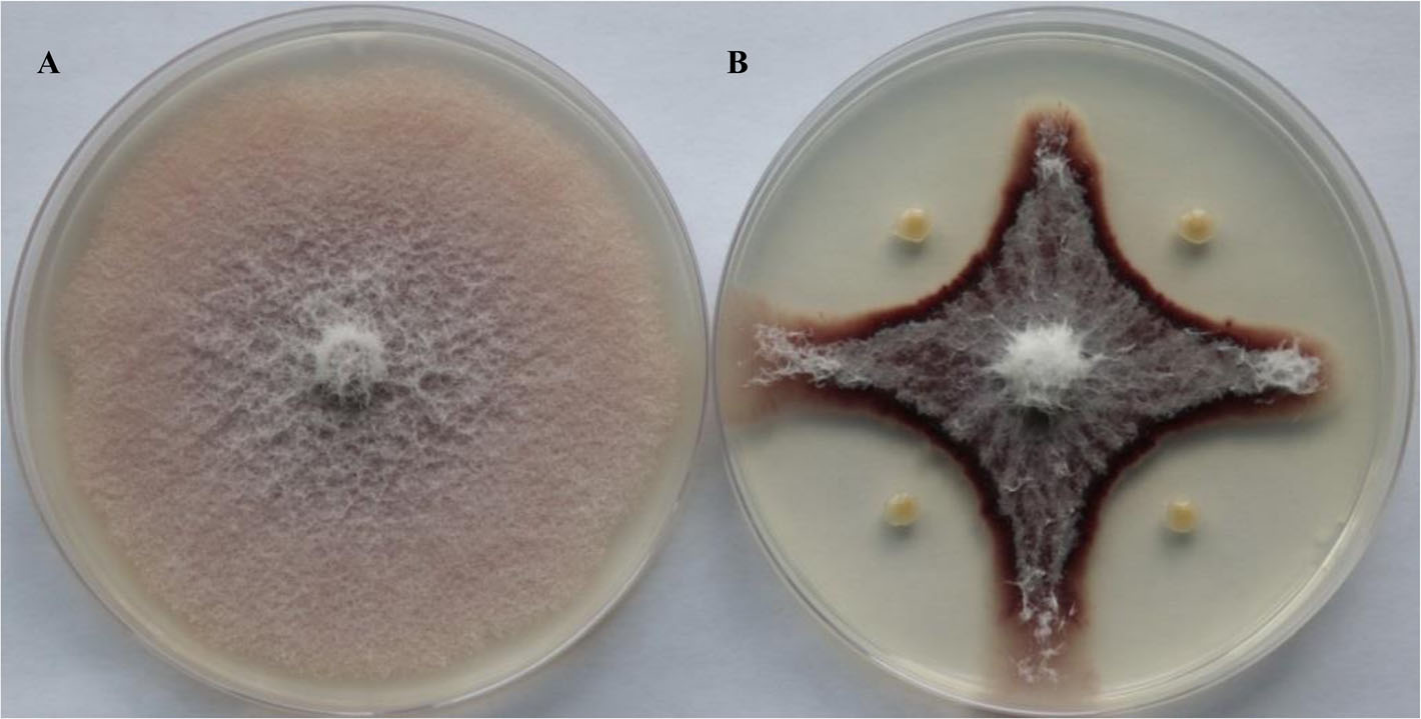
Antagonistic effect of HX-140 against *F. oxysporum*. **a**, the control; **b**, Co-inoculated

A total of 76 bacterial isolates showing inhibitory activities against *F. oxysporum* were isolated from the rhizosphere soil samples of rape, pepper and flax (Table 1). Among them, preliminary tests show that 23 strains showed antagonistic effects against four pathogens (*Rhizoctonia solani* Kuhn, *Phytophthora capsici* Leon., *Sclerotinia sclerotiorum* Lib., and *Fusarium oxysporum* Schl.). In this paper, HX-140 was taken as an example to study the biological control mechanism and the effect of HX-140 on cucumber Fusarium wilt control (Fig. 1).

**Table 1:** 76 strains with antagonistic effect on FOC, and their isolated sources

Number	*Rhizoctonia solani* Kuhn	*Phytophthora capsici* Leon.	*Sclerotinia sclerotiorum* Lib.	*Fusarium oxysporum* Schl.	Rhizosphere soil sources
HX-35	–	***	–	*	Flax
HX-37	***	***	**	**	Pepper
HX-42	***	*	***	***	Pepper
HX-50	**	**	*	**	Pepper
HX-51	**	**	*	**	Pepper
HX-53	**	***	–	**	Pepper
HX-54	–	–	–	**	Pepper
HX-60	**	–	***	**	Rape
HX-61	–	–	*	*	Rape
HX-62	–	–	**	***	Rape
HX-63	–	–	–	**	Rape
HX-64	–	–	–	*	Rape
HX-65	–	*	–	*	Rape
HX-69	–	*	–	*	Rape
HX-70	***	***	–	*	Rape
HX-71	–	*	–	*	Rape
HX-72	***	***	***	**	Rape
HX-73	***	**	***	*	Rape
HX-88	**	**	*	*	Rape
HX-89	*	***	–	*	Rape
HX-91	*	*	–	*	Pepper
HX-92	***	***	***	*	Pepper
HX-94	–	–	–	*	Pepper
HX-99	*	*	–	*	Flax
HX-100	*	**	–	*	Flax
HX-101	–	**	–	*	Flax
HX-105	–	–	–	*	Rape
HX-107	*	**	**	*	Rape
HX-110	**	–	–	**	Pepper
HX-111	**	–	–	**	Pepper
HX-112	–	–	–	**	Pepper
HX-113	–	–	–	**	Pepper
HX-115	**	–	–	*	Pepper
HX-116	**	–	–	*	Pepper
HX-121	***	**	**	*	Flax
HX-122	**	–	–	*	Flax
HX-123	**	*	**	**	Flax
HX-124	**	*	**	*	Flax
HX-125	***	**	***	*	Flax
HX-126	**	**	**	*	Flax
HX-128	**	*	**	**	Flax
HX-129	**	–	*	*	Flax
HX-130	*	–	*	*	Flax
HX-131	*	–	–	*	Flax
HX-133	*	**	–	*	Flax
HX-134	*	–	*	*	Flax
HX-135	**	–	*	*	Flax
HX-136	***	–	***	**	Flax
HX-137	***	**	**	*	Flax
HX-138	***	–	–	*	Flax
HX-139	–	–	**	*	Flax
HX-140	**	**	***	**	Rape
HX-142	*	–	*	*	Rape
HX-143	***	–	*	*	Rape
HX-144	–	–	*	*	Flax
HX-145	**	–	–	*	Flax
HX-146	*	**	–	*	Flax
HX-147	***	**	–	*	Rape
HX-148	**	–	–	*	Rape
HX-149	–	**	–	*	Rape
HX-161	*	*	–	*	Rape
HX-162	*	–	–	*	Rape
HX-163	**	**	**	**	Rape
HX-165	*	*	*	*	Rape
HX-166	*	–	*	*	Rape
HX-167	**	**	*	*	Rape
HX-168	*	***	*	**	Rape
HX-169	**	**	**	*	Rape
HX-172	–	–	–	*	Rape
HX-173	–	–	–	*	Rape
HX-179	*	–	**	*	Rape
HX-180	**	–	**	**	Flax
HX-181	*	–	–	*	Rape
HX-182	*	–	**	*	Rape
HX-184	***	*	**	*	Pepper
HX-187	***	*	–	*	Rape

-: the bacteriostatic zone diameter is zero; *: The antibacterial zone diameter is greater than 0 and less than 10 mm; **: The diameter of the inhibition zone is greater than or equal to 10 mm, and less than 20 mm; ***: The diameter of the inhibition zone is greater than or equal to 20 mm

**Fig. 2:**
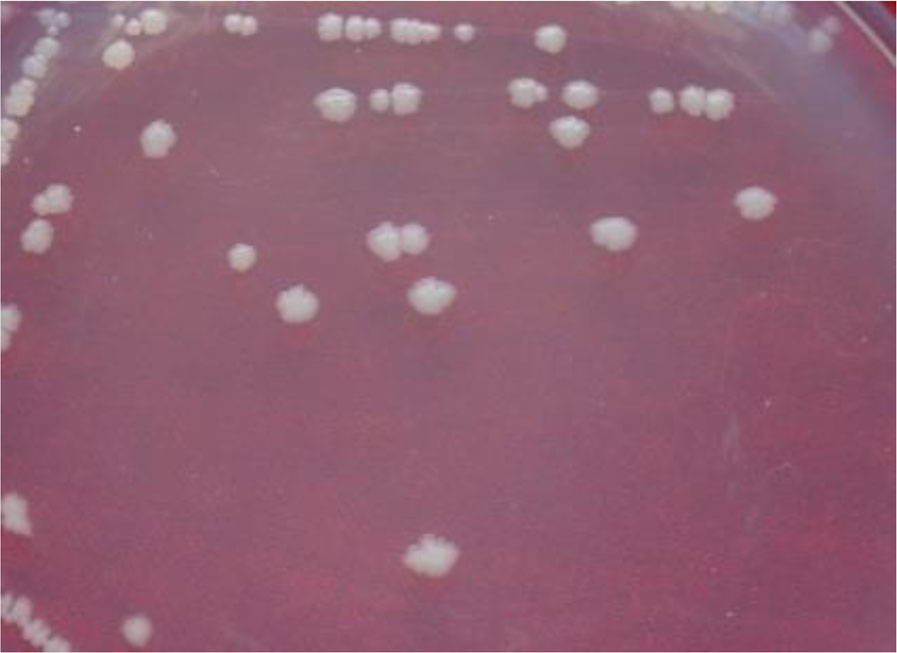
Colony morphology of strain HX-140 on NA medium

Based on DNA sequence of the 16S rDNA gene of strain HX-140 (GenBank accession number MF136611), this strain was revealed to be most similar to *P. polymyxa*. When strain HX-140 was cultured on a NA plate it appeared as small, milky white, opaque colonies with irregular edges. The bulged surface appeared smooth and moist (Fig. 2). Gram staining showed that the strain HX-140 was a rod-shaped, Gram-negative bacterium (Figure S1, see Supplemental materials.).

Compared to the type strain of *Paenibacillus* (*P. polymyxa* ATCC 842^T^), strain HX-140 exhibited almost identical physiological characteristics, except for a variation in nitrate reduction. Strain HX-140 and *P. polymyxa* ATCC 842^T^ are both positive for growing in 3% NaCl (w/v), anaerobic growth, starch hydrolysis, casein hydrolysis, the VP (Voges-Proskauer) test, and acid production using galactose, _L_-arabinose, _D_-fructose, and _D_-xylose. However, HX-140 was negative for indole production and unable to use citrate (Table 2).

**Table 2:** Physiological and biochemical characterization of strain HX-140

Characteristics	***P. polymyxa*** HX-140 ^**a**^	***P. polymyxa*** ATCC 842^**Ta**^
Anaerobic growth	+	+
Growth with 3% NaCl	+	+
Hydrolysis of starch	+	+
Hydrolysis of casein	+	+
Nitrate reduction	+	v
Indole production	–	–
VP test	+	+
Citrate utilization	–	–
Acid production from carbon sources		
Galactose	+	+
_L_-Arabinose	+	+
_D_-Fructose	+	+
_D_-Xylose	+	+

^*a*^*+* Positive reaction, − Negative reaction, *V* variable

**Fig. 3:**
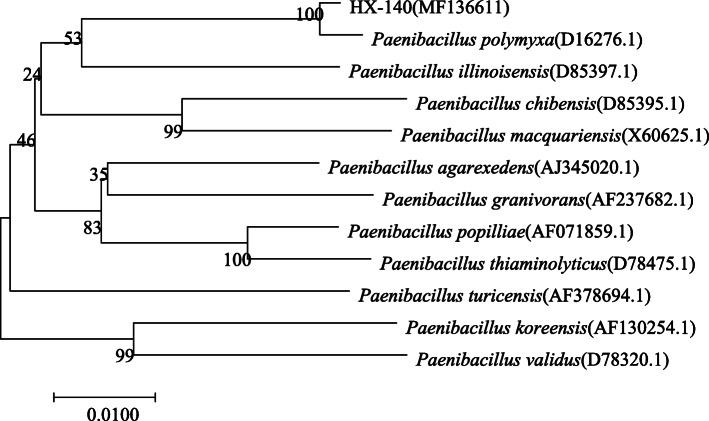
Phylogenetic analysis based on 16S rDNA sequences of strain HX-140 and closely related species constructed by the neighbor-joining method. The number at the nodes indicates the level of bootstrap support (%) based on a 1000 replicates. The scale bar at the bottom indicates the genetic distance. GenBank accession numbers analyzed here are shown in parentheses

Based on the combined morphological, physiological, and molecular characterizations (Fig. 3), strain HX-140 was identified as *P. polymyxa*.

### Evaluation the antifungal activity of HX-140 and its associated antifungal compounds

The antifungal activity of *P. polymyxa* HX-140 on tested pathogens was greater than 60% and had an optimal efficacy of 79.2% against *F. oxysporum* (Table 3), suggesting that HX-140 is a broad-spectrum antagonistic bacterium with biocontrol potential.

**Table 3:** Antifungal activity of strain HX-140 in dual cultures

Fungal pathogens	Diameter of the fungal colony in the test group (mm) ^**a**^	Inhibition rate (%) ^**b**^
*Fusarium oxysporum* Schl.	18.75 ± 0.73 e	79.2 ± 0.8 a
*Fusarium lini* Boll.	31.00 ± 0.38 bc	65.6 ± 0.4 cd
*Colletotrichum lini* West.	28.63 ± 0.38 d	68.2 ± 0.4 b
*Colletotrichum gloeosporioides* Penz.	29.88 ± 0.55 cd	66.8 ± 0.6bc
*Sclerotinia sclerotiorum* Lib.	31.94 ± 0.71 b	64.5 ± 0.8 d
*Phytophthora capsici* Leon.	34.38 ± 0.46 a	61.8 ± 0.5 e
*Rhizoctonia solani* Kuhn	30.38 ± 0.68 bc	66.3 ± 0.8 cd

^a, b^ Data are mean ± standard error. Different letters are significantly different according to Duncan’s multiple range test (*P* < 0.05)

**Fig. 4:**
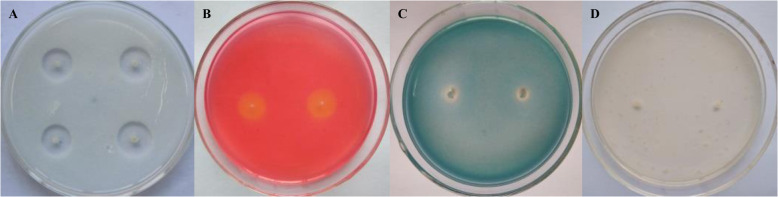
Detection of antagonist protein production by strain HX-140. **a**, skim-milk agar medium; **b**, CMC medium; **c**, Aniline blue agar medium; **d**, chitin-agar medium

*P. polymyxa* HX-140 created clear zones around colonies on the skim-milk agar, CMC, and Aniline blue agar media (Fig. 4), indicating that *P. polymyxa* HX-140 exhibited extracellular protease, cellulase and β-1,3-glucanase activities, respectively. This was not observed on the chitin-agar, indicating the absence of chitinase activity.

**Fig. 5:**
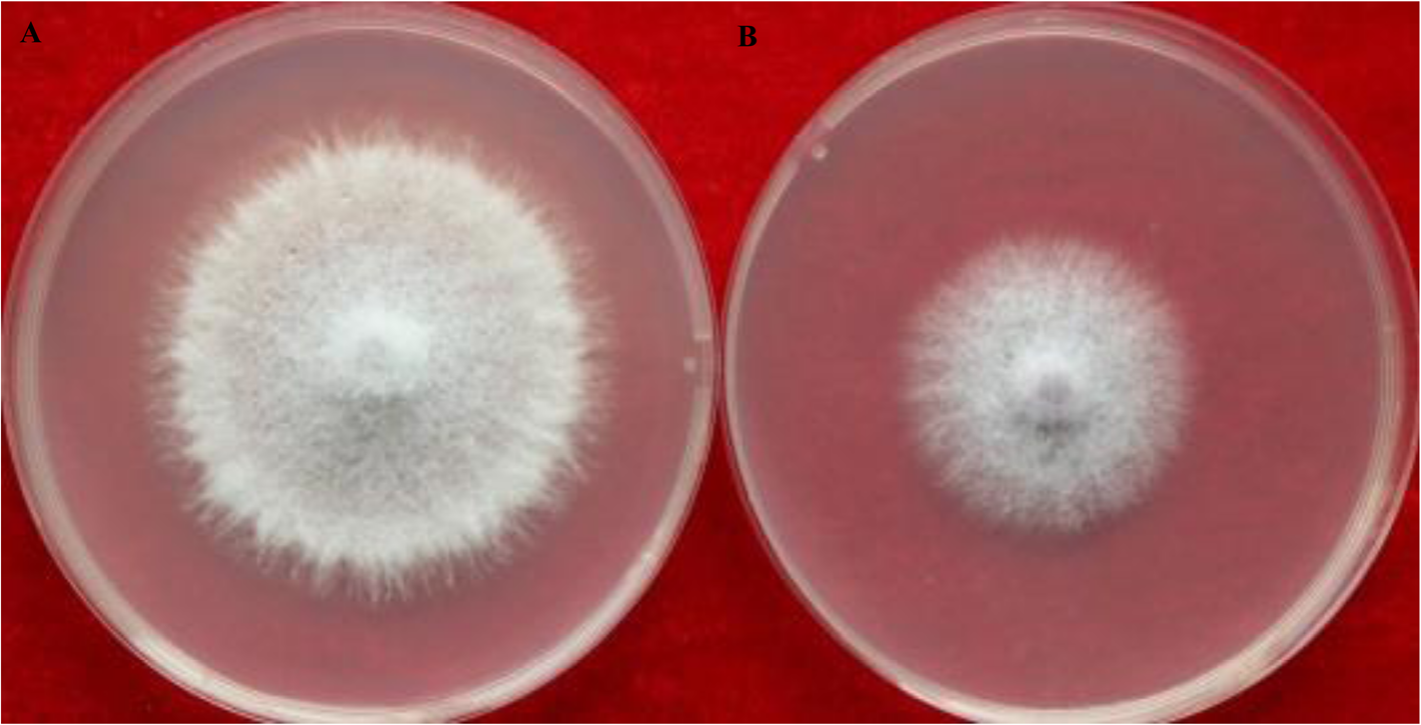
Detection of antifungal organic volatile compounds from strain HX-140. **a**, control group; **b**, test group

In the sealed plate assays, *P. polymyxa* HX-140 exhibited a significant inhibition of the growth of *F. oxysporum* mycelia compared with that in the control group (Fig. 5), indicating that *P. polymyxa* HX-140 was able to produce anti-fungal volatiles.

### Control effect of strain HX-140 on cucumber Fusarium wilt in the greenhouse and field

#### Pot experiment

The biocontrol efficacy of HX-140 was studied with both pot and field experiments. The symptoms of Fusarium wilt appeared 18 days after inoculation with *F. oxysporum*. Strain HX-140 had a positive effect in controlling Fusarium wilt of cucumber in this experiment. The disease index of the T, CK1 and CK2 treatment groups was 20, 11.5 and 45, respectively. The disease index of cucumber seedlings with the *P. polymyxa* HX-140 and *F. oxysporum* treatment (T) was significantly lower than those seedlings with only *F. oxysporum* (CK2). As expected, the healthiest seedlings were in the negative control with only sterile water (CK1). The infection of Fusarium wilt in cucumber seedlings was significantly (*P* < 0.05) reduced to 35%, and the relative control efficacy was 55.6% after treatment with *P. polymyxa* HX-140 (Table 4).

**Table 4:** Inhibitory effect of strain HX-140 on cucumber seedling diseases in the greenhouse pot experiment

Group	Incidence rate (%)^**a**^	Disease index^**a**^	Control efficacy (%)
CK1	15.00 ± 2.89 c	11.50 ± 0.61 b	
CK2	60.00 ± 0.00 a	45.00 ± 4.62 a	55.6
T	35.00 ± 8.66 b	20.00 ± 5.77 b	

*CK1* negative control with only sterile water, *CK2* treatment with only *F. oxysporum*, *T* treatment with both *P. polymyxa* HX-140 and *F. oxysporum*

^a^ The lowercase letters in the table indicate significant differences between treatments(*P* < 0.05)

#### Field experiment

Within the field experiment, a small proportion of cucumber seedlings exhibited symptoms of wilting before the inoculation of the HX-140 bacterial suspension. The field site was partitioned into small plots all with similar rates of infected plants and similar disease indices. These small plots were randomized to receive four treatments: CK, T1, T2 and T3 treatment (Table 5). Fourteen days after inoculation with varying concentrations of HX-140 suspensions, the incidence rate and disease index of the control group was 76.19% and 59.37, respectively. The efficacies of different concentrations (10^6^, 10^7^ and 10^8^ cfu/mL) of HX-140 bacterial suspension were evaluated and shown to be 19.5, 41.1, and 50.9%, respectively. The control efficacy increased as the concentration of HX-140 increased. Likely influenced by a diversity of biotic and abiotic factors in the field, the control efficacy of strain HX-140 in the field was lower than that in the greenhouse pot culture. However, all three treatments (T1, T2, and T3) showed lower disease incidence and lower disease index than those of the control group. These results suggest that *P. polymyxa* HX-140 had a significant suppressive effect on Fusarium wilt of cucumber seedlings in the field with a history of the disease.

**Table 5:** Inhibitory effect of strain HX-140 on cucumber seedling diseases in the field experiment

Group	Before inoculation of HX-140		After inoculation of HX-140		Control efficacy (%)^**a**^
	Incidence rate (%)^**a**^	Disease index^**a**^	Incidence rate (%)^**a**^	Disease index^**a**^	
CK	28.57 ± 1.03 a	7.44 ± 0.68 a	76.19 ± 0.60 a	59.37 ± 0.45 a	
T1	27.38 ± 1.58 a	7.44 ± 1.29 a	68.45 ± 0.59 b	47.77 ± 1.12 b	19.5
T2	27.38 ± 2.15 a	7.44 ± 1.44 a	54.76 ± 1.57 c	34.97 ± 2.43 c	41.1
T3	29.17 ± 0.60 a	7.74 ± 0.52 a	44.65 ± 3.72 d	30.36 ± 1.80 c	50.9

*CK* Control group with no inoculation, *T1* treatment with 10^6^ cfu/mL bacterial suspension of HX-140, *T2* treatment with 10^7^ cfu/mL, *T3* treatment with 10^8^ cfu/mL bacterial suspension of HX-140

^a^ The lowercase letters in the table indicate significant differences between treatments (*P* < 0.05)

## Discussion

In this study, the antifungal activity of *P. polymyxa* HX-140 on *F. oxysporum* was tested in vitro prior to a greenhouse pot experiment, and *P. polymyxa* HX-140 exhibited antagonistic activity against the tested pathogenic fungi. As determined by the greenhouse pot and the field experiments, *P. polymyxa* HX-140 significantly decreased the wilt disease of cucumber seedlings caused by FOC. Specifically, biocontrol efficacies of strain HX-140 in the greenhouse pot and field experiments were 55.6 and 50.9%, respectively, similar to or higher than several previously reported candidate biocontrol agents. For example, the biocontrol efficacies of *Streptomyces albospinus* CT205 were reported to be 8.7 and 51.9% in 2011 and 2012, respectively [39]. In their field experiment, the disease and death incidences were 40.9 and 23.4% respectively in the CK group, and 31.7 and 15.6% after treatment with strain CT205, both of which were lower than that identified for HX-140 in this study. However, the biocontrol efficacy of CT205 in its proprietary formulation (BOF-CT205) was higher than HX-140 in reducing Fusarium wilt [39]. The high biocontrol efficacy of BOF-CT205 may be related to the specialized supplements within the formulation which may enhance the ability of strain CT205 to grow in the field environment and exert antagonistic effects in the soil. It is also possible that the supplemented nutrients in the biocontrol/bio-organic fertilizer stimulated the growth of cucumber roots [39]. Regardless of the potential mechanism (s), the high efficacy of BOF-CT205 suggests several potential avenues for improving the biocontrol effects of strain HX-140 in field applications.

In our study, *P. polymyxa* strain HX-140 was selected due to its high antifungal activity against *F. oxysporum*. In addition, *P. polymyxa* HX-140 displayed a broad spectrum of antifungal activities against six other pathogens. *P. polymyxa* was previously designated to the genus *Bacillus* and reclassified to the new genus *Paenibacillus* in 1994 [10]. *P. polymyxa* strains have been reported as highly effective biocontrol agents for controlling soil borne fungal diseases [26]. For example, Anthracnose symptoms caused by *C. gloeosporioides* and *C. acutatum* in harvested apples were suppressed by 83.6 and 79%, respectively, after treatment with *P. polymyxa* APEC128 [23]. *P. polymyxa* SC09–21 produced cellulase and protease to control *P. capsici*, and the gene expression levels of pathogenesis-related (PR) proteins, including CaPR4 and CaChi2, were also enhanced after treatment with the SC09–21 strain [42]. There have been some studies on the application of *P. polymyxa* in the control of *F. oxysporum*. For example, *P. polymyxa* NSY50 can significantly up-regulate the expression level of defense related genes PR1 and PR5 in cucumber roots at the early stages upon challenge with FOC [13], *P. polymyxa* WLY78 can strongly inhibit fusarium wilt of cucumber [25], NSY50 and WLY78 can improve the resistance of cucumber to FOC, and also have direct control effect on FOC. Some *P. polymyxa* strains were able to inhibit the growth of *F. oxysporum* f. sp. *albedinis* for a long time in vitro [4]. *P. polymyxa* SQR-21 systemically affects root exudates of watermelon to decrease the conidial germination of *F. oxysporum* f.sp. *niveum* [30]. *P. polymyxa* antagonizes the plant pathogenic fungus *F. oxysporum* in liquid medium by means of an interaction process in which the presence of living bacteria is a prerequisite for continuous suppression of fungal growth [11].

We analyzed the potential contributors to the antifungal activities of *P. polymyxa* HX-140 against *Fusarium* spp. *P. polymyxa* HX-140 exhibited strong protease, cellulase and β-1,3-glucanase activities and produced antifungal VOCs. The fungal cell wall is a dynamic and complex structure. For most fungi, the core of the cell wall is a branched β-1,3 and β-1,6 glucan that is linked to chitin via a β-1,4 linkage. The composition of the cell wall varies markedly between different species of fungi [2, 6, 24]. Protease and β-1,3-glucanase are both potent enzymes that degrade fungal cell walls [5, 32], while endoglucanases have been shown to cleave internal β-1,4-glycosidic bonds [40]. We found that VOCs produced by *P. polymyxa* HX-140 inhibited the mycelial growth of FOC in vitro, which indicates that they may play an important role in the inhibition of soil-borne diseases caused by FOC. The antifungal activity of VOCs has often been an understudied component of research on biocontrol agents, including those of *P. polymyxa* strains [36]. VOCs produced by different biocontrol bacteria operate through varying modalities, such as through the suppression of fungal mycelium growth, the induction of plant systematic resistance, plant growth promotion, and through nematicidal activity [10, 15, 21, 43].

## Conclusion

This study focused on the isolation, identification and characterization of the antagonist activity of the bacterium *P. polymyxa* HX-140 against *F. oxysporum* f. sp. *cucumerinum,* the causative agent of fusarium wilt on cucumber. Our in vitro analyses showed the antagonistic effect of HX-140 on pathogen growth, including its potent enzymatic activities against fungal pathogens. HX-140 was shown to reduce the incidence of cucumber wilt disease in the greenhouse. Furthermore, the bacterium reduced the incidence of cucumber wilt disease by 50.9% in the field, consistent with its significant application potential in the field. The potential molecular contributors to its antifungal activity as identified here also lay a foundation for further studies of the genes and biochemical pathways of HX-140 to improve its antagonistic efficiency and commercial application in the field.

## Methods

### Phytopathogenic strains

The fungal strains (*Fusarium oxysporum*, *Fusarium lini*, *Colletotrichum lini*, *Colletotrichum gloeosporioides*, *Sclerotinia sclerotiorum*, *Phytophthora capsici*, and *Rhizoctonia solani*) used in the study were provided by the Key Laboratory of Biology and Processing of Bast Fiber Crops, Ministry of Agriculture, Institute of Bast Fiber Crops, Chinese Academy of Agricultural Sciences. and stored at 4 °C using potato dextrose agar (PDA) medium.

### Isolation and screening of antagonistic bacterial strains

To screen for bacteria with antifungal activities, rhizosphere soil samples of healthy flax and rape crops grown in Gongzhuling City, Jilin Province, China were obtained. The soil samples were suspended and serially diluted 10-fold using sterile water. The diluted suspensions were spread onto Petri plates containing nutrient agar (NA) medium and incubated at 28 °C for 3 days. Three replicates were performed for each dilution. Bacterial isolates were sub-cultured and purified as single colonies. Representative colonies of different sizes and morphologies were stored on NA slants at 4 °C.

All bacterial isolates were tested for their ability to inhibit fungal growth using the technique of dual culture analysis [13, 45]. Three replicates were used in each treatment and the experiments were repeated twice. In the case of inhibition of *F. oxysporum* f. sp. *cucumerinum*, the plates were incubated at 28 °C in the dark and the inhibitory effect on growth was assessed after 9 days. The bacterial isolate with the strongest antagonistic effect was selected for further study.

### Characterization of strain HX-140

#### 16S rDNA analysis

Isolate HX-140, a strain with the highest anti-*Fusarium oxysporum* activity, was obtained from the screening test. In order to taxonomically identify isolate HX-140, its total genomic DNA was extracted using the Bacteria Genomic DNA Kit (CWBIO, Shanghai, China), following the manufacturer’s instructions. The 16S rDNA was amplified using the universal bacterial primers 27f (5′-AGAGTTTGATCCTGGCTCAG-3′) and 1492r (5′-GGTTACCTTGTTACGACTT-3′) [38], as described by Rameshkumar and Nair [34]. The amplicon was purified and sequenced by Sangon Biological Engineering Co., Ltd. (Shanghai, China). The DNA sequence was analyzed by comparing with those in NCBI GenBank using the BLAST program [3]. A phylogenetic tree was constructed using the neighbor-joining method [37] with Molecular Evolution Genetics Analysis (MEGA) software version 7.0.

#### Morphological and physiological features

The morphological characteristics of strain HX-140 on the NA plate were recorded after incubation at 28 °C for 3 days.

Physiological tests were performed following the Systematic Determinative Manual of General Bacteria [12]. Gram staining was observed using an Olympus BX53 microscope (Olympus: Tokyo, Japan; software: cellSens Entry 1.8).

### Antifungal activity assay

To assess the potential antifungal effects of HX-140, antagonistic activity against seven phytopathogenic fungi was assessed on PDA plates by dual culture analysis as described in a previous study [45]. Three replicates were performed for each treatment and the experiments were repeated twice. The plates were incubated for 9 days in the dark at 28 °C and the antagonism was determined by measuring the zone of inhibition, the distance between the growing edges of HX-140 and fungi. The inhibition ratio was calculated using the following formula [22]:

$$ \mathrm{Inhibition}\ \mathrm{rate}\ \left(\%\right)=\left(\mathrm{diameter}\ \mathrm{of}\ \mathrm{fungal}\ \mathrm{colony}\ \mathrm{in}\ \mathrm{control}-\mathrm{diameter}\ \mathrm{of}\ \mathrm{fungal}\ \mathrm{colony}\ \mathrm{in}\ \mathrm{treatment}\right)/\mathrm{diameter}\ \mathrm{of}\ \mathrm{fungal}\ \mathrm{colony}\ \mathrm{in}\ \mathrm{control}\times 100\%. $$

### Analysis of the antifungal compounds of HX-140 against FOC in vitro

Determination of the production of protease, chitinase, cellulase and β-1,3-glucanase.

The production of protease by HX-140 was analyzed using the spot inoculation technique [1] on a skim-milk agar medium containing equal volumes of sterilized 4% solution agar and milk solution [7]. The chitinase production activity of HX-140 was checked on chitin-agar medium containing fine powdered chitin (0.4% w/v [18];). Cellulase activity was assessed on carboxymethyl cellulose (CMC) medium containing NaNO_3_ 2 g, K_2_HPO_4_ 1 g, MgSO_4_·7H_2_O 0.5 g, KCl 0.5 g, FeSO_4_·7H_2_O 0.01 g, and agar 16 g in 1000 ml distilled water at pH 6.8, supplemented with 1% CMC–Na salt as the carbon source [29]. The activity of β-1,3-glucanase was detected on aniline blue agar medium [20, 32]. Petri plates containing blank skim-milk agar, chitin-agar, CMC, and aniline blue agar mediums were used as controls, respectively. All tests were carried out in triplicate, and the whole experiment was repeated twice. The plates were incubated at 28 °C for 3 days.

### Evaluation the antifungal volatile organic compounds of HX-140

The production of volatile organic compounds (VOCs) by strain HX-140 was determined using the sealed plate method [16]. Briefly, HX-140 was streaked on NA medium in the cover of a Petri plate and a 5 mm disk of actively growing *F. oxysporum* mycelia was placed in the center of the PDA medium on the bottom dish. The dish containing the mycelial plug was inverted over the bacterial plate. The dishes were sealed with parafilm. All tests were carried out in triplicate and the experiment was repeated twice. The plates were incubated for 9 days in the dark at 28 °C, and the antagonism was determined by comparing the diameters of *F. oxysporum* colonies in the test groups with the control groups.

### Greenhouse pot experiment

Cucumber seeds (No. 4 Jinchun) were surface-sterilized by immersion in 0.1% mercuric chloride for 60 s, rinsed in sterile water 3 times, and then stored at 4 °C overnight to induce germination. Seeds were germinated in a Petri plate incubated with a piece of moist filter paper in the dark at 28 °C and then sown into the 9 × 9-hole seedling pots and covered with approximately 1 cm of sterilized substrate (121 °C for 20 min) [13, 46]. Cucumber seedlings were grown in a plant growth chamber at 28 °C, 80 ± 10% relative humidity, and 12,000 Lux light intensity with a photoperiod of 14 h light and 10 h dark.

An actively growing *F. oxysporum* mycelia disk was picked from the margin of the dish and inoculated in PDB (Potato Dextrose Broth) liquid medium. The liquid culture was incubated at 28 °C for 48 h on a rotary shaker at 180 r/min and then diluted to 10^8^ cfu/mL using PDB for backup. Actively growing cells of strain HX-140 were incubated in Lysogeny broth (LB) medium at 28 °C for 24 h at 180 r/min and then centrifuged at 12000 r/min for 5 min. The bacteria precipitate was washed three times with sterile water and then diluted to 10^8^ cfu/mL for downstream use.

The pot experiment was carried out in greenhouse using soil inoculated with 10^8^ cfu/g microorganisms (*F. oxysporum* spores and/or strain HX-140 cells in 1 mL, representing about 5% of the weight of soil transplanted with the seedling). Equal volumes of Fusarium liquid culture and HX-140 suspension were mixed with the nutrient soil evenly in the treatment group (T). Control group 1 (CK1) was treated with an equal volume of sterile water, and the control group 2 (CK2) was treated with equal volumes of Fusarium liquid culture and sterile water. All treatments were kept at 28 °C for 2 days to ensure that the Fusarium pathogen (in T and CK2 treatments) and strain HX-140 (in T) antagonist colonized the soil.

For each treatment, twenty cucumber seedlings in the two-leaf stage were planted in the treated nutrient soil. Before planting, their roots were mechanically damaged to help initiate infection. Three replicates were maintained in each treatment and the experiment was repeated twice. The disease severity was investigated 20 days after transplantation of cucumber seedling.

Disease severity of cucumber seedlings was divided into five classes and recorded using a disease index ranging from 0 to 4 as follows:

- Level 0-Plant was healthy with no illness;
- Level 1-Leaves with slight wilting;
- Level 2-Stems and leaves slightly wilted with necrotic spots on the leaves;
- Level 3-Plants moderately wilted with necrotic spots on leaves and stems;
- Level 4-Plants died.

The disease index and biocontrol efficacy were calculated according to the following formulas [47]:

$$ \mathrm{Incidence}\ \mathrm{rate}=\mathrm{the}\ \mathrm{number}\ \mathrm{of}\ \mathrm{diseased}\ \mathrm{plants}/\mathrm{total}\ \mathrm{number}\ \mathrm{of}\ \mathrm{investigated}\ \mathrm{plants}\times 100\% $$

$$ \mathrm{Disease}\ \mathrm{in}\mathrm{d}\mathrm{ex}=\left[\sum \left(\mathrm{the}\ \mathrm{number}\ \mathrm{of}\ \mathrm{disease}\mathrm{d}\ \mathrm{plants}\ \mathrm{in}\ \mathrm{this}\ \mathrm{in}\mathrm{d}\mathrm{ex}\times \mathrm{disease}\ \mathrm{in}\mathrm{d}\mathrm{ex}\right)/\left(\mathrm{total}\ \mathrm{plants}\ \mathrm{in}\mathrm{vestigated}\times \mathrm{highest}\ \mathrm{disease}\ \mathrm{in}\mathrm{d}\mathrm{ex}\right)\right]\times 100. $$

$$ \mathrm{Biocontrol}\ \mathrm{efficacy}=\left[\left(\mathrm{disease}\ \mathrm{index}\ \mathrm{of}\ \mathrm{control}\ \mathrm{group}\ 2-\mathrm{disease}\ \mathrm{index}\ \mathrm{of}\ \mathrm{treatment}\ \mathrm{group}\right)/\mathrm{disease}\ \mathrm{index}\ \mathrm{of}\ \mathrm{control}\ \mathrm{group}\ 2\right]\times 100\%. $$

### Field experiment on cucumber seedlings

The field experiments were carried out on an experimental farm of the Institute of Bast Fiber Crops, Chinese Academy of Agricultural Sciences, in Yuanjiang Country (28°42′26″ N, 112°14′37″ E), Hunan Province, China. The total field plot size was ~ 200 m^2^, divided into subplots of 1.4 × 1.6 m. The chosen field for this experiment was planted with cucumber for more than 3 years, and the incidence of cucumber wilt was over 70%. We used a complete randomized block design with three replicates for each treatment. Each sub-plot was planted with 56 seedlings. The experiment was repeated twice from June to November 2018.

The field trial included four treatments: (i) control group with no inoculation (CK); (ii) a treatment group inoculated with 10^6^ cfu/mL of the HX-140 bacterial suspension (T1); (iii) a treatment group inoculated with 10^7^ cfu/mL of the HX-140 bacterial suspension (T2); and (iv) a treatment group inoculated with 10^8^ cfu/mL of the HX-140 bacterial suspension (T3). When the seedlings had 1–2 leaves, 20 mL of HX-140 bacterial suspension was used to drench their roots. The incidence of cucumber wilt among the different treatments was investigated before HX-140 inoculation and 14 days after inoculation.

The disease severity of cucumber seedlings was recorded using a disease index ranging from 0 to 4, identical to the greenhouse pot experiment, and the control efficacy was calculated using the following formulas [17, 41]:

$$ \mathrm{Disease}\ \mathrm{in}\mathrm{d}\mathrm{ex}=\left[\sum \left(\mathrm{the}\ \mathrm{number}\ \mathrm{of}\ \mathrm{disease}\mathrm{d}\ \mathrm{plants}\ \mathrm{in}\ \mathrm{this}\ \mathrm{in}\mathrm{d}\mathrm{ex}\times \mathrm{disease}\ \mathrm{in}\mathrm{d}\mathrm{ex}\right)/\left(\mathrm{total}\ \mathrm{plants}\ \mathrm{in}\mathrm{vestigated}\times \mathrm{highest}\ \mathrm{disease}\ \mathrm{in}\mathrm{d}\mathrm{ex}\right)\right]\times 100. $$

$$ \mathrm{Biocontrol}\ \mathrm{efficacy}=\left[1-\left(\mathrm{disease}\ \mathrm{index}\ \mathrm{of}\ \mathrm{treatment}\ \mathrm{group}\ \mathrm{after}\ \mathrm{inoculation}\times \mathrm{disease}\ \mathrm{index}\ \mathrm{of}\ \mathrm{control}\ \mathrm{group}\ \mathrm{before}\ \mathrm{inoculation}\right)/\left(\mathrm{disease}\ \mathrm{index}\ \mathrm{of}\ \mathrm{treatment}\ \mathrm{group}\ \mathrm{before}\ \mathrm{inoculation}\times \mathrm{disease}\ \mathrm{index}\ \mathrm{of}\ \mathrm{control}\ \mathrm{group}\ \mathrm{after}\ \mathrm{inoculation}\right)\right]\times 100\%. $$

### Statistical analysis

Statistical analysis was performed using Microsoft Excel 2010 (Microsoft Corporation, Redmond, WA, USA) and DPS 7.05 software (Zhejiang University, Hangzhou, China). Mean values were compared using Duncan’s multiple range test with *P* < 0.05 as the level of significance.

## Supplementary Information

**Additional file 1: Figure S1** Gram staining of strain HX-140. Bar = 20 μm.

## Data Availability

The datasets generated and/or analysed during the current study are available in the Treebase repository [http://purl.org/phylo/treebase/phylows/study/TB2:S27431], including a total of 12 sequences, and the evolutionary tree was built based on these sequences. The accession numbers of all sequences in NCBI are: AF071859.1, AF130254.1, AF237682.1, AF378694.1, AJ345020.1, D16276.1, D78320.1, D78475.1, D85395.1, MF136611.1 and X60625.1.
