# Supplementary material for: Isolation and characterization of antagonistic Paenibacillus polymyxa HX-140 and its biocontrol potential against Fusarium wilt of cucumber seedlings
Source: BMC Microbiol. 2021 Mar 6;21:75. doi: 10.1186/s12866-021-02131-3 (PMC7936408; doi:10.1186/s12866-021-02131-3)

Isolation and characterization of antagonistic *Paenibacillus polymyxa* HX-140 and its biocontrol potential against Fusarium wilt of cucumber seedlings

Yang Zhai^1^, Jiu-xiang Zhu^1,2^, Tai-meng Tan^1,2^, Jian-ping Xu^1^, Ai-rong Shen^2,3^, Xie-bin Yang^1,2^, Ji-lie Li^2^, Liang-bin Zeng^1*^, Lin Wei^4*^

^1^ Institute of Bast Fiber Crops, Chinese Academy of Agricultural Sciences, Changsha, 410205, China

^2^ Hunan Provincial Key Laboratory of Forestry Biotechnology, Central South University of Forestry and Technology, Changsha, 410004, China

^3^ Hunan Academy of Forestry, Changsha, 410004, China

^4^ Institute of Plant Protection, Hunan Academy of Agricultural Sciences, Changsha, 410125, China

^*^ Correspondence: Dr. Liang-bin Zeng, (zengliangbin@caas.cn), Institute of Bast Fiber Crops, Chinese Academy of Agricultural Sciences, No.348 Xianjiahu West Road, Changsha, Hunan, 410205, China.

Dr. Lin Wei, (nkyweilin@163.com), Institute of Plant Protection, Hunan Academy of Agricultural Sciences, No.726 Yuanda 2nd Road, Changsha, Hunan, 410125, China.

**Figure S1** Gram staining of strain HX-140. Bar=20 μm.


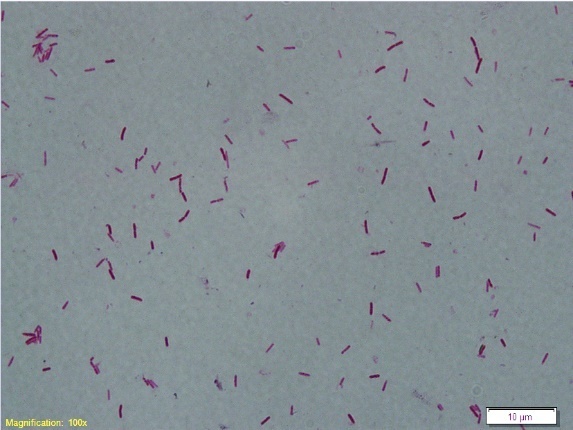

Supplement: Supplementary file 1 — Additional file 1: Figure S1 Gram staining of strain HX-140. Bar = 20 μm. [file 12866_2021_2131_MOESM1_ESM.docx]
